# Drug-resistant *Streptococcus pneumoniae* and Methicillin-resistant *Staphylococcus aureus* Surveillance^1^

**DOI:** 10.3201/eid0910.030454

**Published:** 2003-10

**Authors:** Leigh Ann Hawley, Scott K. Fridkin, Cynthia G. Whitney

**Affiliations:** * Centers for Disease Control and Prevention, Atlanta, Georgia, USA

The Centers for Disease Control and Prevention (CDC) convened a conference on March 12–13, 2003, in Atlanta, Georgia, to discuss improving state-based surveillance of drug-resistant *Streptococcus pneumoniae* (DRSP) and methicillin-resistant *Staphylococcus aureus* (MRSA). The Council of State and Territorial Epidemiologists, the Association of Public Health Laboratories, and CDC co-sponsored the conference; 120 participants from 38 states attended. The conference was organized by the Divisions of Healthcare Quality Promotion and Bacterial and Mycotic Diseases, National Center for Infectious Diseases, CDC. Goals of the meeting included 1) reviewing the rationale for surveillance of DRSP and MRSA, 2) presenting scientific studies highlighting valid and meaningful methods of performing state-level surveillance, 3) sharing state-level surveillance experiences, and 4) identifying unmet needs of the state health departments in performing such surveillance.

The primary theme of the conference general sessions was the public health impact of DRSP and MRSA and the need for accurate surveillance data to track and monitor resistance trends. *S. pneumoniae* is a major cause of respiratory infections in the United States. Since the early 1990s, the prevalence of resistance to single and multiple antibiotics has been increasing in pneumococci. Antimicrobial drug resistance in *S. pneumoniae* can vary among populations and is influenced by local prescribing practices and the prevalence of resistant clones. Conference presenters discussed the role of surveillance in raising awareness of the resistance problem and in monitoring the effectiveness of prevention and control programs. National- and state-level epidemiologists discussed the benefits of including state-level surveillance data with appropriate antibiotic use programs designed to address the antibiotic prescribing practices of clinicians. The potential for local surveillance to provide information on the impact of a new pneumococcal vaccine for children was also examined; the vaccine has been shown to reduce infections caused by resistance strains

Since the early 1990s, *S. aureus* infections resistant to oxacillin (MRSA) have increased steadily. Several scientists reported two recent changes in the epidemiology of MRSA: its emergence in persons without established risk factors and the emergence of vancomycin-resistant *S. aureus* (VRSA). These new developments underscore the need for scientifically valid, yet financially feasible, state-based surveillance that will aid in understanding the changing epidemiology of MRSA disease. Such understanding will allow effective implementation of MRSA prevention and control programs. Prevention programs will differ on the basis of which populations are most affected (long-term care, community, and hospice). Current programs focus on reducing cross-transmission through improved hand hygiene and wound care. (More information is available from: URL: http://www.cdc.gov/ncidod/hip/Aresist/aresist.htm).

Conference participants generally agreed that a lack of resources for surveillance has challenged state public health agencies committed to monitoring emerging antimicrobial drug resistance. A conclusion drawn from conference sessions was that statically sound methods of data collection that capture valid, meaningful, and useful data and meet the financial restrictions of state budgets are indicated.

Active, population-based surveillance for collecting relevant isolates is considered the standard criterion. Unfortunately, this type of surveillance is labor-intensive and costly, making it an impractical choice for many states. The challenges of isolate collection, packaging and transport, data collection, and analysis may place an unacceptable workload on laboratory and epidemiology personnel.

Epidemiologists from several state health departments that have elected to implement enhanced antimicrobial drug-resistance surveillance programs presented alternative surveillance methods currently implemented in their states. Several surveillance models and knowledge gained by state-based epidemiologists provided key insights into the challenges and benefits of implementing enhanced surveillance programs.

Two methods frequently used by states are sentinel (i.e., survey of subset of laboratories) and antibiogram (i.e., cumulative susceptibility data) surveillance. Common difficulties were identified with implementing sentinel systems. Those difficulties included logistical obstacles with isolate or data processing and communication breakdowns between laboratory, epidemiology, and hospital infection control personnel. Care must be taken in selecting the numbers and types of laboratories to participate in the sentinel network States collecting antibiograms from hospitals and state laboratories also face challenges, including incompatible formatting of drug-testing panels, the inconsistent inclusion of duplicate or repeat isolates, and inconsistent reporting of denominator data. Solutions to these problems commonly involve improving communication between clinical microbiology laboratories and state health departments, including laboratory input in decision making and providing feedback of data from the system to participants. Guidance for aggregating cumulative susceptibility data (i.e., antibiograms) has been published and can serve as a guide for states and clinical microbiology laboratories in conducting surveillance. Also, having designated staff was essential for successfully implementing most programs. Presenters agreed that the benefits of collecting local data from these systems are substantial and will assist prevention programs.

Another aspect of surveillance focuses on detecting rare events. Such reports may include new changes in susceptibility, new mechanisms of resistance, susceptibility of unusual pathogens, and unexpected sources of resistant organisms. Establishing good communication among personnel in health departments and clinical laboratories is important for improving reporting of such events.

Allocating resources for improved surveillance is considered a practical and responsive step for states interested in tracking local resistant trends. Local data are important for raising public awareness, establishing resources and prevention activities, developing and informing treatment guidelines, monitoring trends, and motivating behavior change among clinicians.

This meeting and ongoing efforts to study and validate surveillance methods will assist local health authorities in making decisions programs to monitor antimicrobial drug resistance. The 2-day conference provided an opportunity to initiate an exchange of current practices and knowledge gained among states and territories. This process marks the first phase in building networks that may potentially enhance training resources, provide guidance for program development, and identify further technical assistance needed from CDC.

